# Supplementary material for: Unexpected Arabinosylation after Humanization of Plant Protein N-Glycosylation
Source: Front Bioeng Biotechnol. 2022 Feb 18;10:838365. doi: 10.3389/fbioe.2022.838365 (PMC8894861; doi:10.3389/fbioe.2022.838365)
Supplement: Supplementary file 1 [file DataSheet1.docx]

**Supplementary Material**


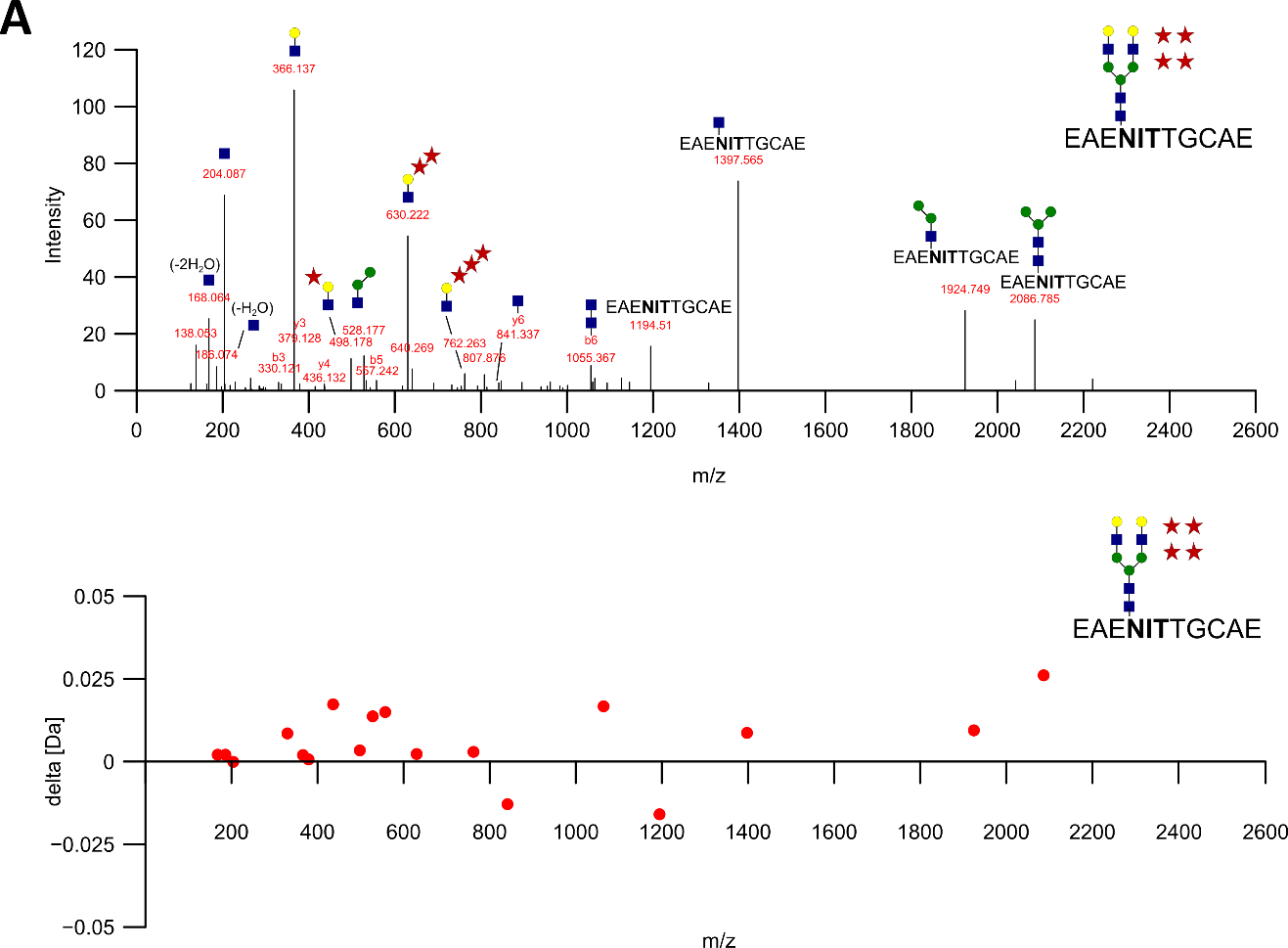


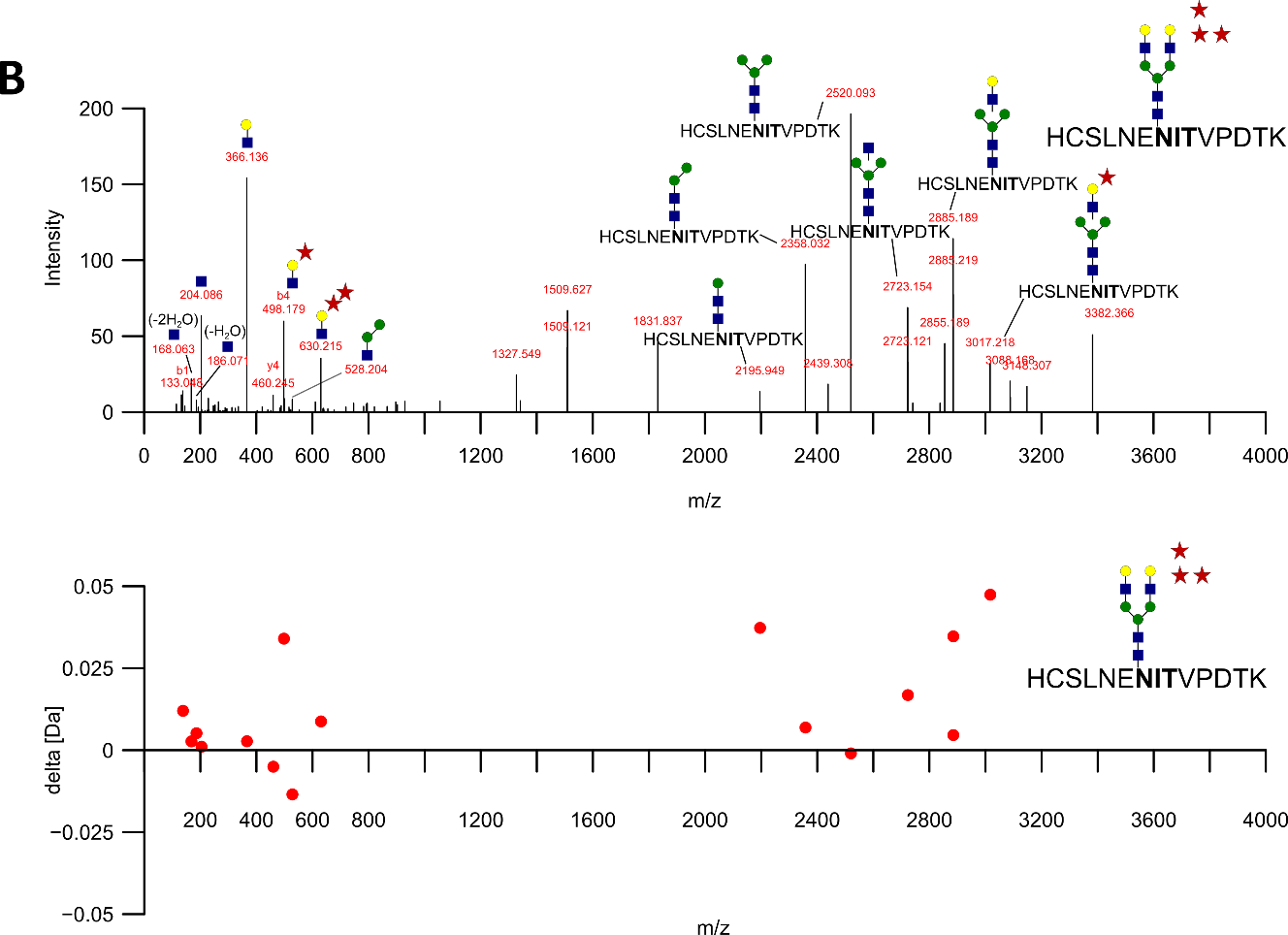


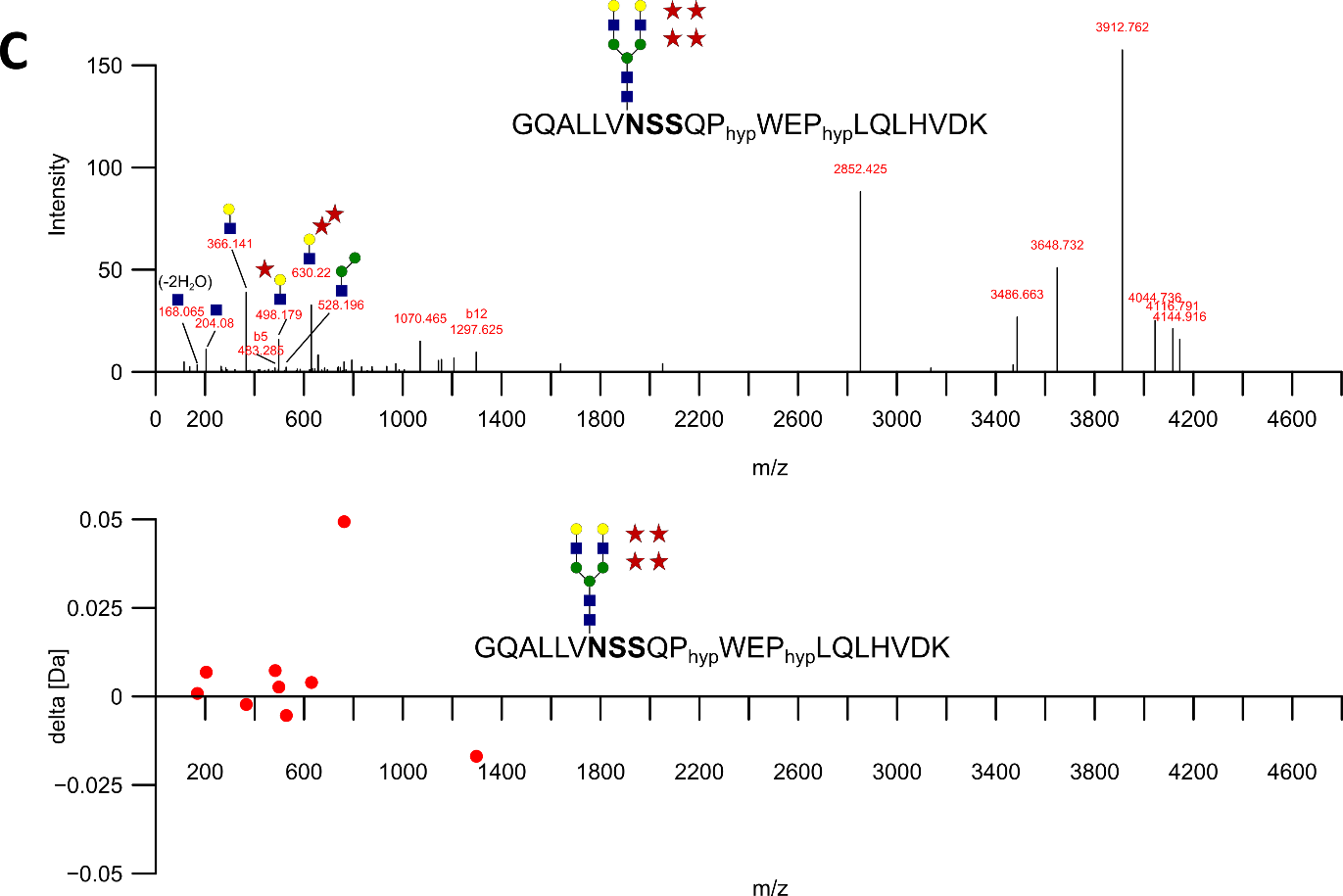


**SUPPLEMENTARY FIGURE S1. Collision-induced dissociation (CID) fragment spectra of di-antennary galactosylated and additionally pentosylated glycopeptides representing the three rhEPO N-glycosylation sites (Asn24, Asn38, Asn83). (A)** CID fragment spectrum of the identified glycopeptide EAE**NIT**TGCAE ([M^+^3H^+^]^+^ = 1115.7574). The identified precursor mass corresponds to a di-antennary galactosylated N-glycan with four attached pentoses. **(B)** CID fragment spectrum of the identified glycopeptide HCSLNE**NIT**VPDTK ([M^+^4H^+^]^+^ = 912.3763). The identified precursor mass corresponds to a di-antennary galactosylated N-glycan with three attached pentoses. **(C)** CID fragment spectrum of the identified glycopeptide GQALLV**NSS**QP_hyp_WEP_hyp_LQLHVDK ([M^+^4H^+^]^+^ = 1136.2589). The identified precursor mass corresponds to a di-antennary galactosylated N-glycan with four attached pentoses. The mass shift of carbamidomethylation (+57.0214 Da) is added to all contained cysteine residues. Hydroxylation of a proline is indicated by P_hyp_ (+15.9949 Da). Spectra were acquired on a Q-TOF instrument from a sample of line X24. The monoisotopic masses of the detected sugar reporter ions are as followed: [GlcNAc]^+^ (blue square) = 204.0867, [GlcNAc - H_2_O]^+^ = 186.0761, [GlcNAc - 2H_2_O]^+^ = 168.0655, [GlcNAcHex]^+^ = 366.1395, [GlcNAcHex_2_]^+^ = 528.1923, [GlcNAcHexPent]^+^ = 498.1818, [GlcNAcHexPent_2_]^+^ = 630.2241; [GlcNAcHexPent_3_]^+^ = 762.2664 with Hex= Hexose = galactose (yellow circle) or mannose (green circle) and Pent = pentose (red star). N-glycosylation consensus sequences of depicted glycopeptides are shown in bold. Below each fragment spectrum the distribution of the mass errors of identified fragments is shown.


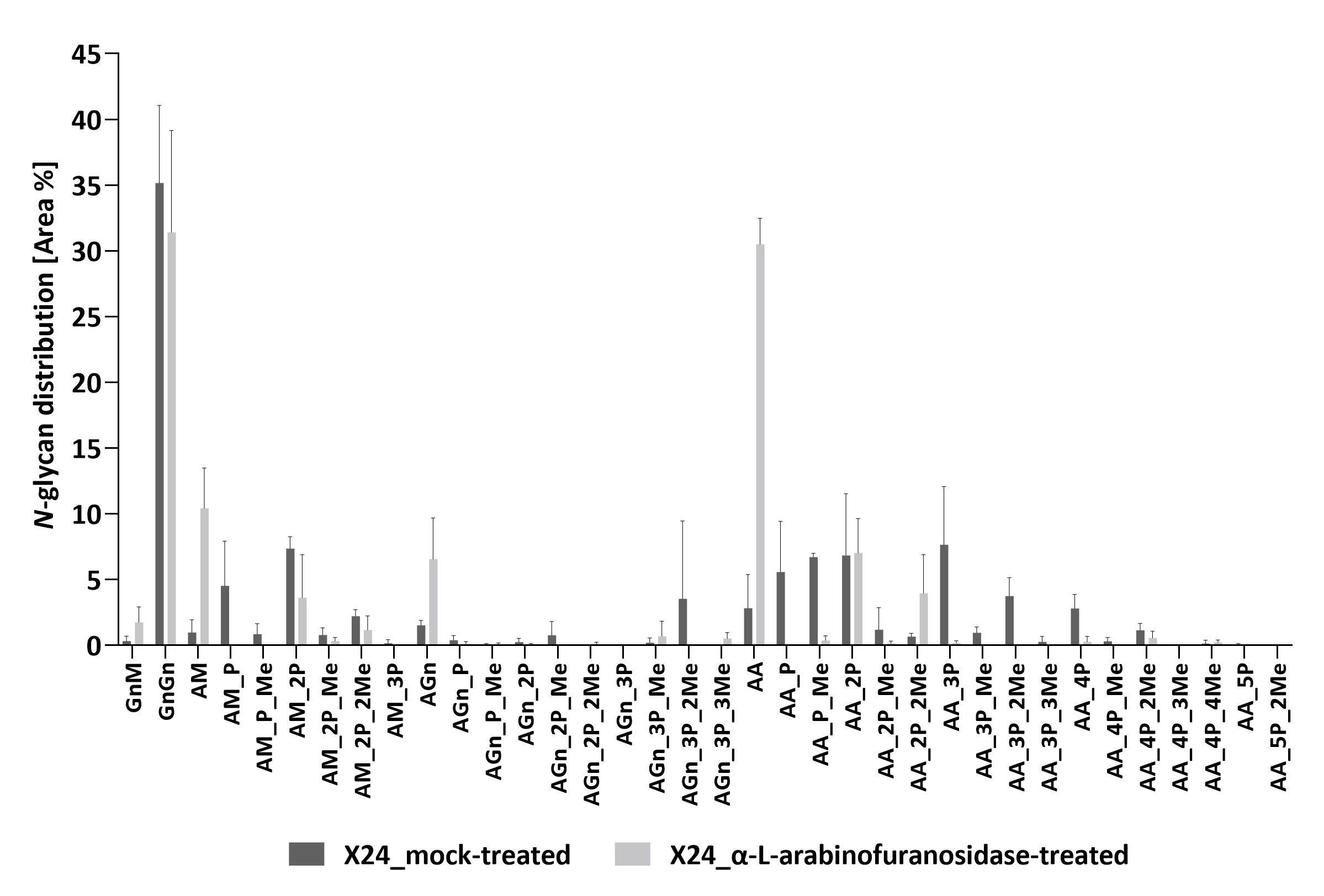


**SUPPLEMENTARY FIGURE S2. Quantitative MS/MS analysis of the N-glycan distribution on rhEPO from α-L-arabinofuranosidase treated vs. mock-treated samples.** Prior to MS analysis rhEPO-containing samples of line X24 were digested with α-L-arabinofuranosidase, while mock-treated samples without enzyme addition were prepared in parallel. For MS analysis trypsin and GluC-released rhEPO glycopeptides were analyzed. The N-glycosylation pattern of α-L-arabinofuranosidase-treated and mock-treated glycopeptides is represented as relative percentages of all identified N-glycan structures across all three N-glycosylation sites. P: Pentose, Me: Mass increment of 14.0157 Da corresponding to methylation. The mean of three replicates with standard deviation is depicted.
